# *In Vivo* Intracellular Oxygen Dynamics in Murine Brain Glioma and Immunotherapeutic Response of Cytotoxic T Cells Observed by Fluorine-19 Magnetic Resonance Imaging

**DOI:** 10.1371/journal.pone.0059479

**Published:** 2013-05-08

**Authors:** Jia Zhong, Masashi Sakaki, Hideho Okada, Eric T. Ahrens

**Affiliations:** 1 Department of Biological Sciences and Pittsburgh NMR Center for Biomedical Research, Carnegie Mellon University, Pittsburgh, Pennsylvania, United States of America; 2 Department of Neurological Surgery, University of Pittsburgh School of Medicine, Brain Tumor Program, University of Pittsburgh Cancer Institute, Pittsburgh, Pennsylvania, United States of America; University of Nebraska Medical center, United States of America

## Abstract

Noninvasive biomarkers of anti-tumoral efficacy are of great importance to the development of therapeutic agents. Tumor oxygenation has been shown to be an important indicator of therapeutic response. We report the use of intracellular labeling of tumor cells with perfluorocarbon (PFC) molecules, combined with quantitative ^19^F spin-lattice relaxation rate (R_1_) measurements, to assay tumor cell oxygen dynamics *in situ*. In a murine central nervous system (CNS) GL261 glioma model, we visualized the impact of Pmel-1 cytotoxic T cell immunotherapy, delivered intravenously, on intracellular tumor oxygen levels. GL261 glioma cells were labeled *ex vivo* with PFC and inoculated into the mouse striatum. The R_1_ of ^19^F labeled cells was measured using localized single-voxel magnetic resonance spectroscopy, and the absolute intracellular partial pressure of oxygen (pO_2_) was ascertained. Three days after tumor implantation, mice were treated with 2×10^7^ cytotoxic T cells intravenously. At day five, a transient spike in pO_2_ was observed indicating an influx of T cells into the CNS and putative tumor cell apoptosis. Immunohistochemistry and quantitative flow cytometry analysis confirmed that the pO_2_ was causally related to the T cells infiltration. Surprisingly, the pO_2_ spike was detected even though few (∼4×10^4^) T cells actually ingress into the CNS and with minimal tumor shrinkage. These results indicate the high sensitivity of this approach and its utility as a non-invasive surrogate biomarker of anti-cancer immunotherapeutic response in preclinical models.

## Introduction

Malignant gliomas are the most common type of primary brain tumor and a significant public health problem, with more than 14,000 new cases diagnosed each year in the US [1]. Glioblastoma multiforme (GBM) is by far the most common and most malignant of the glial tumors. Patients diagnosed with GBM have a median survival of approximately 3 months if untreated. Surgical removal is generally the first stage of treatment followed by radiation and/or chemotherapy. Recent clinical trial results show that concomitant temozolomide and radiotherapy improved the 2-year survival to 27.2% compared to only a 10.2% survival rate for patients receiving radiation therapy alone [2]. Although this trial demonstrates a significant advancement in the treatment of GBM, novel strategies are required to better treat patients with this aggressive cancer.

Immunotherapy using live cells has opened up new avenues for targeting brain tumors with minimal damage to healthy tissues [3], [4], [5], [6], [7]. We recently demonstrated that following intravenous transfer of *ex vivo* activated tumor-specific Tc1, but not Tc2, cytotoxic CD8+ T cells traffic to CNS tumor sites and mediate a potent CNS anti-tumor response [8]. However, key questions remain unanswered regarding the mechanisms of immune cell entry and function in the CNS.

The successful development of novel immunotherapeutic agents hinges upon finding biomarkers that are able to monitor the therapeutic response *in vivo*. Often, disease biomarkers involve accessible tissue samples such as blood; however, for cell therapies, an imaging biomarker may be preferable to confirm cell delivery to the target site, and potentially provide a non-invasive tool for the therapeutic response. In the early development of preclinical cancer therapeutic candidates, significant curative outcomes or tumor shrinkage may not occur. Nonetheless, sensitive surrogate biomarkers are needed to confirm therapeutic delivery to the tumor cells and provide real-time feedback about any incremental anti-tumoral efficacy.

Tumor oxygenation, an important aspect of tumor physiology, has been shown to correlate with tumor angiogenesis, recurrence, and malignant progression [9]. Measurement of tumor oxygenation may provide new avenues for the development of novel therapies such as hypoxia-activated pro-drugs and hypoxia-specific gene therapy [10]. More importantly, tumor oxygen levels can be a critical factor determining the tumor response to radiation and chemotherapy [11], [12]. Thus, noninvasive measurements of tumor intracellular partial pressure of oxygen (pO_2_) may have profound implications to further our understanding of tumor biology and in the development of advanced cancer therapies.

Prior work from our laboratory has established PFC cell labeling and tracking methods using ^19^F magnetic resonance imaging (MRI) [13]. In this approach, isolated cells of interest are labeled *ex vivo* using a PFC emulsion designed for uptake by non-phagocytic cells. Following transfer to the subject, labeled cells are tracked *in vivo* using fluorine-19 (^19^F) MRI with high specificity for the labeled cells. Building on these cell tracking technologies, a logical extension is to exploit known oxygen-sensing properties of the PFC molecules inside the cell.

PFC emulsions have previously been used to measure pO_2_ *in vivo* using MRI techniques [14], [15], [16], [17], [18], [19]. The PFC molecule perfluoro-15-crown-5 ether (PCE), with twenty fluorine atoms having an equivalent chemical shift, is a molecule that is well suited for ^19^F magnetic resonance spectroscopy (MRS) and MRI. PCE dissolves paramagnetic oxygen, thereby decreasing the ^19^F spin-lattice relaxation rate (R_1_) [14], [20], [21]. The ^19^F R_1_ of PCE is linearly proportional to the oxygen concentration in proximity to emulsion droplets. Tissue oxygenation *in vivo* can be determined using a standard calibration curve correlating PCE relaxation rates to pO_2_ [22].

In this study, we aimed to detect and characterize putative tumor pO_2_ changes that occur following glioma/T cell interactions. Our approach uses intracellular labeling of glioma cells with PFC *ex vivo* prior to implantation into the CNS [13]. In established tumors, immunotherapy was performed using intravenously infused MHC-matched, glioma antigen-reactive CD8+ T cells from Pmel-1 mice [23]. Immunohistochemistry (IHC) and flow cytometry analysis were used to further confirm our *in vivo* findings. These data show that an increase in pO_2_ can reliably be observed, even though relatively few T cells actually ingress into the CNS tumor. Overall, these results show that these non-invasive intracellular oximetry methods have high sensitivity and cell specificity.

## Materials and Methods

### Pmel-1 mouse-derived cytotoxic T cells

All animal protocols were approved by the Carnegie Mellon University and/or University of Pittsburgh institutional animal care and use committee (IACUC). All mice received humane care in compliance with the *Guide for the Care and Use of Laboratory Animals* published by the National Institute of Health. T cells were obtained from splenocytes of Pmel-1 mice (Jackson laboratories, Bar Harbor, ME). Pmel-1 mice are transgenic for a T cell receptor (TCR) recognizing human glioma antigen hgp100_25–33_ [23]. The hgp100-specific TCR allows for targeting of tumor cells expressing the cross-reactive murine gp100_25–33_ epitope on the major histocompatibility complex H-2D^b^. The CD8+ T cells were enriched from Pmel-1 splenocytes using magnetic cell sorting (Miltenyi Biotec, Boston, MA) and stimulated with the hgp100_25–33_ peptide (5 µg/ml) and 100 U/ml recombinant human interleukin-2 (rhIL-2) in the presence of irradiated (3,000 rad) splenocytes from C57BL/6 mice (Jackson Laboratories) used as feeder cells. At 48 hours after initial stimulation, the cells were re-stimulated under the same conditions and harvested on day 7. The CD8+ T cells for control experiments were isolated from wild-type C57BL/6 mouse splenocytes using magnetic cell sorting (Miltenyi Biotec) and stimulated *in vitro* using 100 U/ml rhIL-2 and 5 µg/ml CD3 antibody.

For IHC assays, control T cells were labeled with the fluorescent dye, carboxyfluorescein succinimidyl ester (CFSE, Vybrant cell tracer kit, Molecular Probes Inc., Eugene, OR) after stimulation. The cells were co-incubated at 37°C with 5 ml of 10 µM CFSE in PBS. After 15 min, the cells were washed and then incubated in complete cell medium with RPMI 1640 supplemented with 10% heat-inactivated fetal bovine serum, 100 units/ml penicillin, 100 µg/ml streptomycin, and 10 mmol/l L-glutamine (Life Technologies Inc., Grand Island, NY) at 37°C for 30 min prior to intravenous injection to the animals.

### *Ex vivo* PCE labeling of GL261 cells

The mouse GL261 glioma cell line was kindly provided by Dr. Prins (University of California at Los Angeles). This cell line expresses the human glioma antigen hgp100_25–33_ and produces aggressive tumors in syngeneic mice [24]. The GL261 cell line was maintained in complete cell medium in a humidified incubator at 5% CO_2_ and 37°C.

GL261 glioma cells were labeled *ex vivo* with PCE emulsion (CS-580, Celsense, Inc., Pittsburgh, PA). Briefly, 90% confluent GL261 cells in a 10 cm culture dish were co-incubated with 7.5 mg/ml of PCE emulsion in serum-free media using similar materials and methods described previously [13]. Cell viability was examined after the labeling using the trypan blue exclusion assay and determined to be >90%. After 4 hours of incubation at 5% CO_2_ and 37°C, the cells were washed three times in PBS prior to inoculation.

To measure the PCE content of labeled cells, ^19^F nuclear magnetic resonance (NMR) was used. Labeled cells (1×10^6^) were pelleted in a 5 mm NMR tube and 200 µL of 1% trifluoroacetic acid (TFA) was added as a standard. The sample temperature was maintained at 37°C, and NMR spectroscopy was performed at 11.7 T using a Bruker spectrometer (Bruker Biospin, Billerica, MA) with a recycle delay time of 8 s and 32 averages. The spectral peaks of PCE and TFA were integrated to calculate the mean ^19^F content per cell as previously described [25].

### Glioma model

Female C57BL/6 mice (n = 16), 6–8 weeks old (Jackson Laboratories) were anesthetized with a cocktail of ketamine (Sigma Inc., St. Louis, MO) at 40–95 mg/kg and Xylazine (Lloyd Inc., Shenandoah, IA) at 5–20 mg/kg. Using a stereotaxic instrument, two holes were drilled in the skull 1 mm anterior and 2.3 mm lateral to either side of the bregma using a surgical drill. PCE labeled GL261 glioma cells (5×10^5^) suspended in PBS were inoculated into the right striatum (day 0). Unlabeled GL261 cells (0.5×10^6^) were injected into the left striatum. At day 3, CD8+ T cells (2×10^7^) from Pmel-1 mice were injected intravenously via tail vein (n = 7) using a PBS vehicle. In the control T cell group (n = 4), the same number of CD8+ T cells from wild-type C57BL/6 mice were used. A second control group did not receive any T cell treatment (n = 5).

### Calibration of pO_2_ response

**Figure 1:**
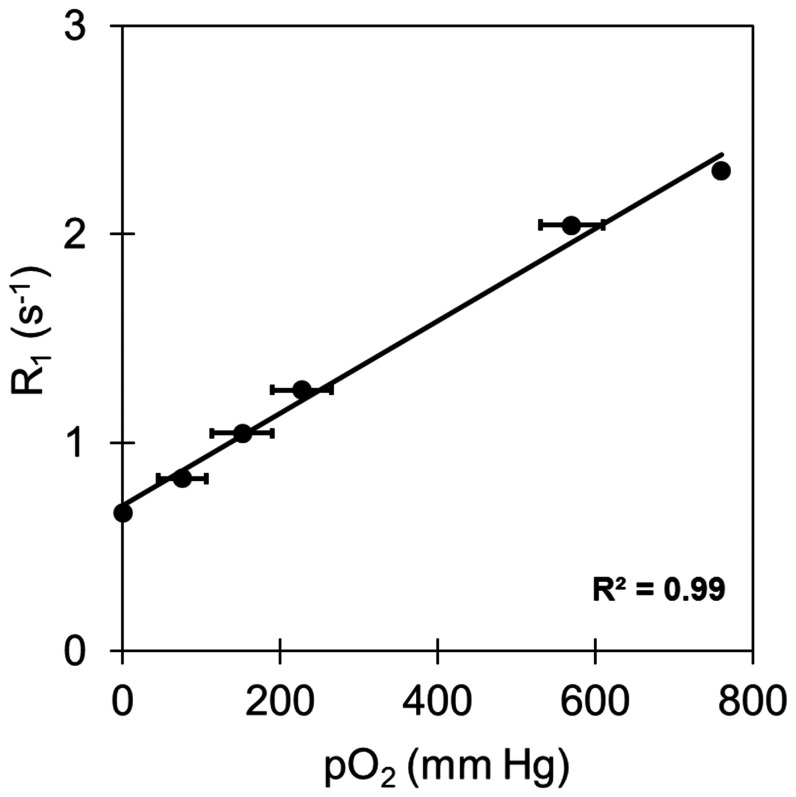
*In vitro* calibration curve for R_1_ = 1/T_1_ versus pO_2_. Here, solid circles represent average ^19^F R_1_ measurements of six oxygen partial pressure values, each in triplicate, ranging from 0% to 100% oxygen in a saturated O_2_/N_2_ mixture of PFC emulsion in water. The solid line is the calibration curve fit by the linear least squares method (R^2^ = 0.99). Data were collected at 11.7 T and 37°C. 100% oxygen is equivalent to 760 mm Hg. (The R_1_ error bars are smaller than the point size.)

The response of R_1_ = 1/T_1_ to pO_2_ for PCE [26] was determined from emulsion samples held at different oxygen pressures, ranging from 0 to 760 mm Hg_._ These samples were prepared by bubbling mixtures of O_2_ and N_2_ gasses for 15 minutes in NMR tubes, and then the tubes were sealed. Samples were prepared in triplicate for each pO_2_ value. The ^19^F R_1_ was measured for each sample at 37°C using an 11.7 T Bruker NMR spectrometer and a saturation-recovery sequence pulse sequence [25]; each recovery curve was fit to a mono-exponential equation. The R_1_ values for triplicate pO_2_ samples were averaged. Data were fit using a linear least square method to yield a linear relationship between R_1_ and pO_2_ given by (Fig. 1, R^2^>0.99)
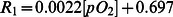
(1)


### *In vivo* ^19^F MRI

Animals underwent ^19^F/^1^H MRI longitudinally after tumor implantation at days 3, 5, 7, and 12. MRI experiments were performed using a Bruker 11.7 T vertical-bore micro-imaging system. The animals were anesthetized with 0.9% isoflurane in 67% O_2_ and 33% N_2_O and maintained at 37°C during the experiments. A reference capillary with a 10% diluted PCE emulsion in 2% agarose was placed close to animal's head in the image field of view (FOV). The ^1^H MRI images were acquired to provide anatomical information of the brain using a spin-echo sequence using the following parameters: repetition time (TR)/echo time (TE) = 700/12 ms, number of averages = 2, slice thickness = 2 mm, number of slices = 8 slices, FOV = 4×4 cm^2^, and matrix size = 256×256. Co-registered ^19^F images with the same FOV and slice thickness were acquired using the rapid acquisition with relaxation (RARE) sequence; the parameters were: TR/TE = 1200/12 ms, RARE factor = 8, number of averages = 128, and matrix size = 64×64 and scan time ∼20 min. The longitudinal loss of the total ^19^F signal in the tumor volume calculated from the total integrated tumor signal, normalized to both the signal of the calibrated external reference and the initial tumor ^19^F signal measured at day 3; the image analysis was performed using the Voxel Tracker software program (Celsense). The ^19^F R_1_ values were measured using a saturation-recovery point resolved spectroscopy (PRESS) sequence, with a single voxel encompassing the entire tumor mass (voxel size = 8×8×8 mm^3^). Twelve TR values were used to measure the R_1_ relaxation rate, ranging between 0.15 and 10 s (total acquisition time ∼50 min). The R_1_ was calculated by integrating the ^19^F spectral peak acquired at different TR values, and the resulting values were fit using a three-parameter single exponential equation [19]. The mean pO_2_ of the tumor cells was then calculated using Eq. [1].

### IHC analysis

Two days after T cell infusion, additional animals (n = 3) were sacrificed for IHC staining. Anesthetized animals were perfused transcardially first with PBS to remove blood, followed by 4% paraformaldehyde (PFA) in PBS. The intact brain tissue was dissected from the skull and stored in 4% PFA overnight. The fixed brain was paraffin embedded and sliced into 8 µm sections encompassing the tumor injection site. Brain tissues were stained by Vectashield hard-set mounting medium with 4′,6-diamidino-2-phenylindole (DAPI) (Vector Laboratories Inc., Burlingame, CA).

### Fluorescence activated cell sorter (FACS) analysis

To quantitate tumor infiltrating lymphocytes (TILs), additional animals received Pmel-1 (n = 3) or wild-type (n = 2) T cells three days after tumor inoculation. Two days after T cell infusion, animals were perfused with PBS through the left cardiac ventricle and sacrificed. Brain tissues were mechanically minced, re-suspended in 70% Percoll solution (Sigma-Aldrich, St. Louis, MO), overlaid with 37 and 30% Percoll, and centrifuged for 20 min at 500×*g*. Enriched TIL populations were recovered at the 70–37% Percoll interface. TILs from animals in the same group were pooled together. The wild-type T cells were stained with fluorescent dye-conjugated antibodies, including: anti-CD3 (17A2), anti-CD8 (53-6.7), and anti-CD4 (GK1.5) (eBioScience Inc., San Diego, CA). For Pmel-1 T cell quantification, we used the H-2D (b)/KVPRNQDWL/hgp100_25–33_ tetramer (National Institute of Health tetramer core facility at Emory University). FACS data were obtained using a BD Accuri C6 flow cytometer (Ann Arbor, MI) and analyzed using Venturione software (Applied Cytometry, Sheffield, UK).

### Statistical analysis

All measurements are presented as mean ± standard deviation (SD). The R_1_ and pO_2_ in three different animal groups, i.e., antigen-specific Pmel-1 T cells, wild-type T cells, and control, were compared by one-way analysis of variance (ANOVA). If there were statistical differences, multiple pairwise comparisons were performed using Tukey's test with a confidence interval of 95%. P values less than 0.05 were considered statistically significant.

## Results

### NMR of GL261 cell pellets

The average fluorine content per GL261 cell was determined to be ∼2.5×10^12^ fluorine atoms/cell using ^19^F NMR analysis [25]. The minimum cell detection sensitivity for ^19^F cell tracking at a comparable level of cell loading is on the order of 10^3^–10^4^ cells per voxel [13]. The intracellular localization of PCE droplets was confirmed using confocal microscopy in 9L tumor cells [25]. Because there are a large number of PCE droplets, on the order of 10^4^, following potential mitosis *in vivo*, daughter cells should contain approximately equal amounts of PCE. Internalization of PCE emulsion droplets had minimal effect on the cell viability *in vitro* via trypan blue exclusion (data not shown).

### *In vivo* MRI of pO_2_

**Figure 2:**
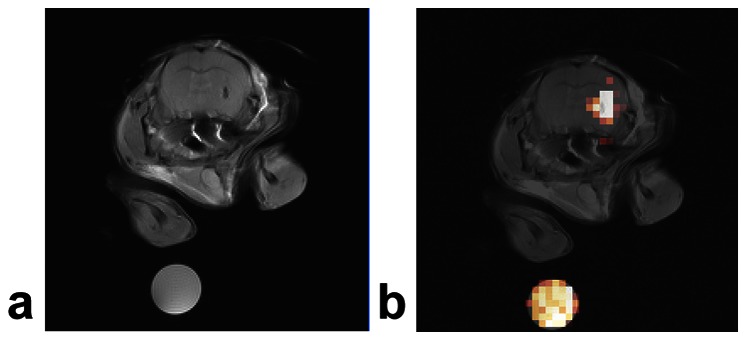
*In vivo* ^19^F/^1^H MRI of mouse glioma showing ^19^F labeled tumor cells. Panel (**A**) is a ^1^H axial image and panel (**B**) is a composite ^19^F and ^1^H image of PCE labeled GL261 glioma cells in the right striatum at day 5 post tumor inoculation. The ^19^F image is rendered in a hot-iron intensity scale, and the ^1^H is in gray scale. Unlabeled GL261 cells were injected into the contralateral striatum in the same imaging plane. Diluted PCE emulsion (9.8 mg/ml) in 2% agarose was used as a ^19^F reference (shown at bottom).

Figure 2 shows ^19^F/^1^H MRI images of glioma cells at day 5 after tumor implantation. A solid tumor is visible in the right striatum in the T_2_-weighted ^1^H image, which co-localizes with the ^19^F ‘hot-spot’ image. No ^19^F signal was detected on the contralateral side of the striatum inoculated with unlabeled cells. The peak ^19^F image signal-to-noise ratio in the tumor was ∼35.

**Figure 3:**
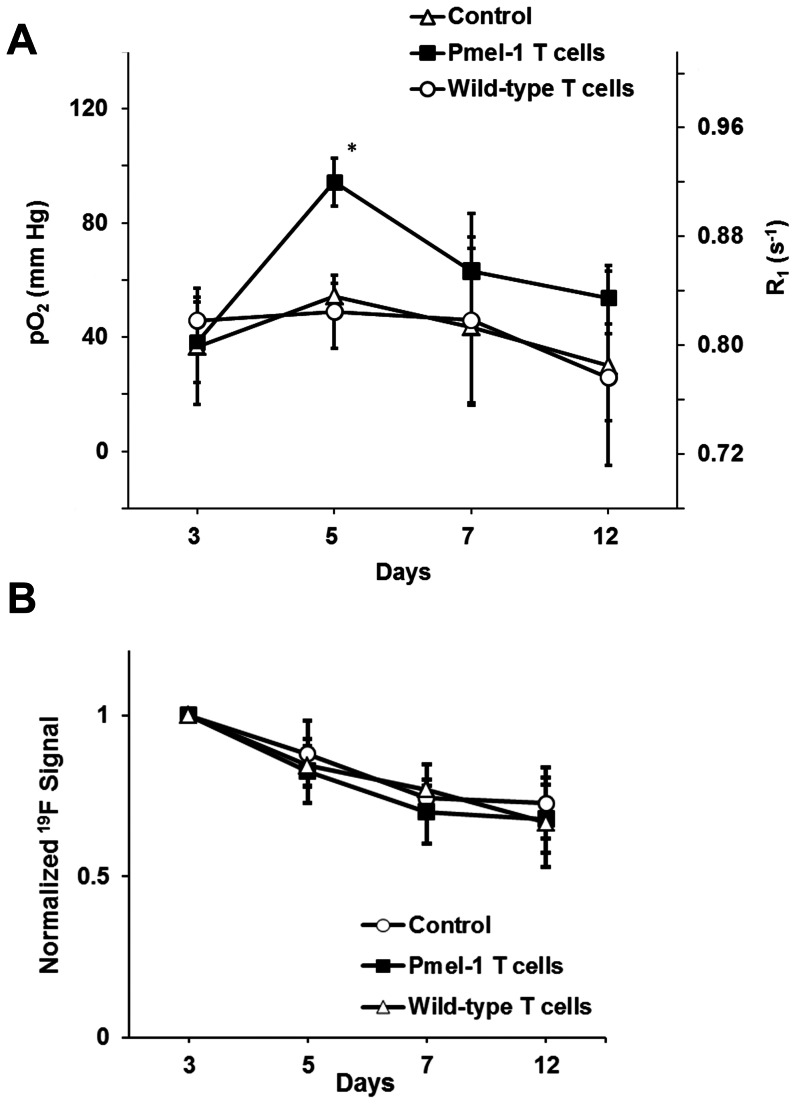
*In vivo* longitudinal pO_2_ changes of CNS GL261 tumor. Panel (**A**) shows the pO_2_ (left axis) and R_1_ (right axis) values. Panel (**B**) displays the total normalized ^19^F signal of the tumor over time. On day 3, Pmel-1 antigen-specific CD8+ T cells or wild-type T cells were injected into the corresponding groups, and the control group received no cells. Here, (*) denotes p<0.05 comparing Pmel-1 group with wild-type T cell and no-cell groups.

Figure 3A (left axis) displays the longitudinal dynamics of tumor pO_2_ in the glioma *in vivo*, with and without the infusion of immunotherapeutic T cells. Notably, approximately two days after Pmel-1 T cell injection, the tumor cells exhibited a significant increase in average R_1_ value (0.92±0.02 s^−1^) when compared to wild-type T cell (0.83±0.03 s^−1^) infusion and (no cell) control (0.84±0.01 s^−1^) groups (Fig. 3A, right axis, p<0.05). The increased R_1_ reveals pronounced elevation of oxygen tension inside the glioma cells (Fig. 3A) when Pmel-1 specific T cells were infused compared to either wild-type T cell infusion or no-cell control groups (94.3±8.4 mm Hg versus 49±13 mm Hg and 54.4±4.4 mm Hg, respectively, p<0.05). We observed that the pO_2_ increase is short-lived, and after this transient spike, tumor pO_2_ in the Pmel-1 T cell group gradually decreased and remained comparable to the other groups beyond seven days after tumor implantation (p = NS). The observed net ^19^F signal decreased longitudinally (Fig. 3B), presumably due to dilution due to cell division and cell death. We note that the PCE agent is not degraded by the cell nor exocytosed [13].

### *Ex vivo* analysis

**Figure 4:**
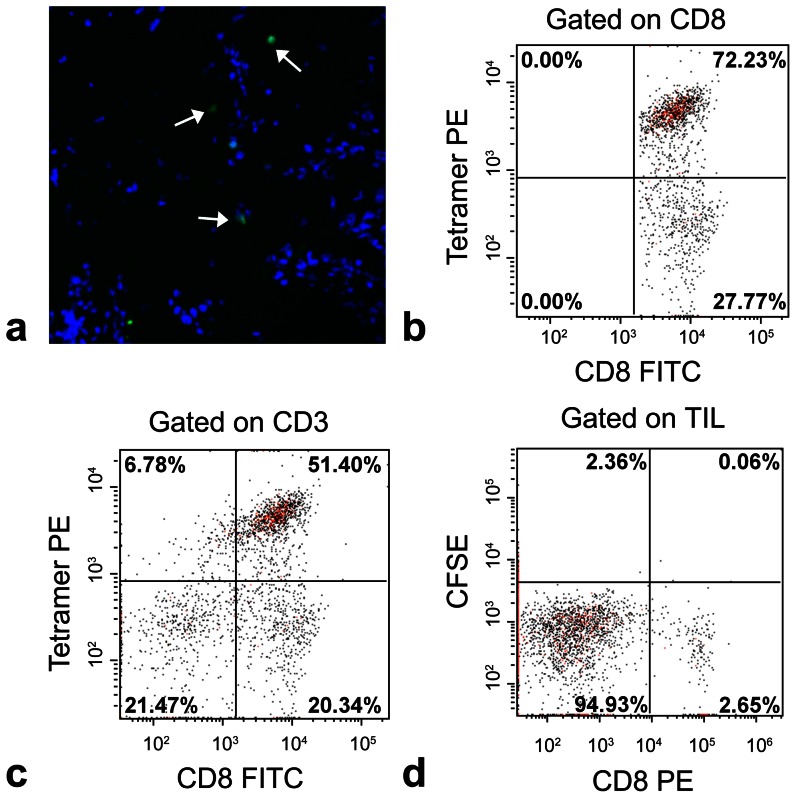
*Ex vivo* analysis shows the infiltration of CD8+ T cells to brain and glioma. Panel **A** shows IHC of fluorescently labeled Pmel-1 CD8+ T cells. Animals were infused with T cells (2×10^7^) and sacrificed for IHC staining two days after cell infusion. Sparse T cells are shown at the arrows. Panels **B–C** show quantitative flow cytometry of Pmel-1, hgp100-specific T cells in brain cell suspension with CD8+ (**B**) and CD3+ (**C**) gating. Panel **D** shows quantitative flow cytometry of wild-type T cells. Here, PE = Phycoerythrin, FITC = Fluorescein isothiocyanate, and TIL = tumor infiltrating lymphocytes.

IHC in excised brain tissue confirmed (Fig. 4A) that Pmel-1 CD8+ T cells infiltrated the GL261 tumor two days after cell infusion, when the oxygen spike was observed *in vivo*. Figure 4A shows a detectable, but sparse, distribution of Pmel-1-specific T cells inside the tumor mass. A single cell suspension was prepared from whole-brain and analyzed using quantitative FACS analysis methods [27]. In the brain cell suspension, Pmel-1-specific T cells could be detected by FACS (Fig. 4B–C), where the average number (n = 3) of cells was approximately 44,000 per brain (n = 3), and each brain contained two Gl261 tumors. In the control group, where wild-type CD8+ T cells were infused, significant T cell trafficking into the brain was not observed via FACS (Fig. 4D).

## Discussion

This study demonstrates, for the first time, the dynamics of the tumor intracellular pO_2_ *in vivo* following treatment with TILs. Overall, these data show that the increase in tumor pO_2_ can reliably be observed using MR techniques, even though relatively few T cells actually ingress into the CNS tumor. We hypothesize that the magnitude and duration of the pO_2_ spike could provide an early surrogate marker of anti-tumor activity.

Immunotherapeutic approaches have emerged as a new avenue for malignant glioma treatment. Taking advantage of the body's own defense system, immunotherapeutic strategies specifically target tumor cells without damaging the surrounding healthy tissue [7], [28]. Existing immune therapy regimes can be categorized into three major types, i.e., general immunomodulation [29], [30], cancer vaccines [5], [31], [32], and adoptive cell transfer (ACT) [3], [33]. In ACT therapies, the TILs are harvested from the body, expanded and stimulated *ex vivo*, then transferred back to the host to elicit an immune response against malignancies. This technique has been used and shown to be effective in eradicating melanomas, and recent efforts have focused on applying ACT to other cancer forms including malignant glioma [7]. In our study, two days after ACT therapy, a transient ‘spike’ in tumor oxygenation was observed. This improvement in tumor intracellular pO_2_ was associated with the infiltration of cytotoxic T cells from circulation (Figs. 4A–C). However, the elevated pO_2_ decayed after two days, which may indicate that the small number of Pmel-1-specific CD8+ T cells that ingress into the tumor were not sufficient to provide a sustained increase in pO_2_, nor obvious tumor shrinkage. In contrast, other studies using similar MRI methods [25] in a 9L glioma model in the rat brain showed a sustained pO_2_ elevation after a single infusion of the potent chemotherapeutic agent bis-chloroethylnitrosourea (BCNU).

Future efforts need to be directed at improving ACT to exert a persistent tumor killing effect, for example, as detected by the pO_2_ response. When combined with systemic IL-2 administration, it has been show that ACT effectively suppressed tumor growth in glioma-bearing rats [3]. It was also reported that cytokine and toll-like receptor-3 agonist (Poly-ICLC) facilitate ACT trafficking to the tumor site, thus helping to kill tumor cells and prolong the survival of glioma-bearing animals [34]. We note that this same study [34] reported the same order of magnitude of CD8+ T cells (∼10^4^) entering the CNS tumor as the present study. Selection of a subset of CD8+ T cells with memory potential and optimization of *in vitro* stimulation conditions for ACT also have been shown to help mediating tumor clearance [35]. Further, depletion of immune response suppressor cells, i.e., regulatory T cells, facilitates the tumor-killing process [36]. The evaluation of these next-generation preclinical experiments using the sensitive surrogate pO_2_ markers described herein is the subject of future work.

Tumor oxygenation is a key factor determining tumor growth, metastasis, and the response to treatment. We detected a moderate decreasing trend in the mean tumor pO_2_ in the control group with tumor growth (Fig. 3A), which echoes the findings in previous studies [17]. In response to the TIL therapy, an increase in oxygenation was observed (Fig. 3A). The increase was commensurate with the appearance of Pmel-1 T cell trafficking to the tumor site, as confirmed by IHC and FACS analysis (Figs. 4A–C). The exact mechanism(s) for the elevation in pO_2_ is unknown. These infiltrated cytotoxic T cells have a TCR that recognizes gp100, thereby inducing tumor apoptosis [37]. We speculate that tumor cells undergoing apoptosis likely have altered intracellular pO_2_ levels. Alternatively, another possibility is that when tumor cells were killed, the PCE emulsion was released to the extracellular space and exposed to a higher pO_2_ or taken up by resident phagocytic cells. However, detailed histological studies in a rat glioma model using labeled 9L cells show that the PCE from apoptotic cells tends to clear and disperse from tissue over time. There is the possibility that a small amount of observed PCE is extracellular from dead cells before the material it is cleared, and this is a limitation of the technique. Theoretically, other factors may influence the ^19^F R_1_ and/or intracellular pO_2_, including arterial pO_2_, cerebral blood flow and perfusion [38], metabolic processes [39], anesthetics [40], and body temperature. In our study, all animals were mechanically ventilated and maintained under constant anesthetic conditions and temperature regulated at 37°C during imaging. Similar experiment settings yielded a stable arterial pO_2_ level in rat [25], thus we believe that we have minimized the confounding effects of arterial pO_2_, anesthetics, and body temperature.

In our study, the pO_2_ was measured by assuming uniform oxygenation in a relatively small solid tumor. The baseline tumor pO_2_ measured in our study was in the similar range as previous studies [41], but lower than that in normal cerebral tissue [15] indicating the presence of hypoxia in an early-stage tumor mass. Hyperoxic gas (67% O_2_ and 33% N_2_O) was used during MRI study. Therefore, the absolute pO2 values are not physiologic values. Tumor oxygenation is likely heterogeneous within a tumor mass, but our current measurements were not designed to capture spatial heterogeneity [17], [38]. Future studies that examines the spatial distribution of pO_2_ using T_1_ mapping methods may further our understanding of pO_2_ changes and its role in tumor physiology.

*Ex vivo* labeling of GL261 was used instead of *in situ* labeling in this study. In previous studies, the PCE emulsion was introduced i.v., or injected directly intratumoral [14], [16], [18], [19]. For i.v. delivery, generally a large PCE dose is injected systemically and, at best, only a small fraction of the PCE is delivered to the tumor tissues, which may be visible several days post-administration. The PCE emulsion droplets are sequestered predominantly in the periphery of the tumors because of the leakage in the tumor vasculature [17], [18], or phagocytosis by tumor-associated macrophage [14], [16]. The above routes of administration generally result in very non-uniform distribution of PCE emulsion deposits in the tumors, and the pO_2_ sensing may be from an ill-defined cell type or extracellular. *Ex vivo* labeling has the advantage of uniform labeling of tumor cells, and daughter cells contain similar amount of PCE after cell division. *Ex vivo* labeling also ensures specificity to tumor cells. It is not possible to use the techniques described herein with spontaneous tumor models or with unlabeled implanted tumor models. Also, the technique is best suited for tumors that are relatively young, i.e., prior to a large number of cell divisions to ensure a relatively uniform PFC distribution. Moreover, inhomogeneity in cell division is possible, which may result in some regions falling under the ^19^F detection threshold and thus not being represented in the overall pO_2_ level.

In this study, an oxygen sensitive PFC compound, PCE, was used for the measurement of pO_2_. PCE has a single ^19^F NMR peak and is a sensitive molecule for MRI and displays high sensitivity to pO_2_ changes (Fig. 1). Other PFC compounds for oximetry including hexafluorobenzene (HFB) [42] and perfluorooctylbromide (PFOB) [43] have been discussed in the literature [19], [21]. We note that the particular PCE emulsion formulation that was used (CS-580, Celsense, Inc.) is designed to be taken up by any cell type in culture regardless of its phagocytic properties and without the use of transfection agents, thus any (cancer) cell type can be labeled.

The most widely used technique to measure tumor pO_2_ is the polarographic oxygen electrode method [9], [44]. Electrode measurements are invasive and several penetrations are required to sample the tumor volume; furthermore this technique is not suitable for longitudinal study of deep-seated tumors. Non-invasive imaging methods for detecting tumor hypoxia are reviewed elsewhere [45] and include positron emission tomography, electron paramagnetic resonance (EPR) imaging, and using near-infrared spectroscopy. Of these imaging methods, only EPR using implantable micron-sized crystalline probes can provide a quantitative measurement of pO_2_.

Overall, in this study, a preclinical ^19^F MRI method was developed to monitor rodent tumor oxygen dynamics *in vivo* associated with adoptive immunotherapy. The potential applications of our methods are not limited to tumor models. It may also be valuable to monitor cellular metabolism in different cell types where intracellular oxygenation plays a critical role, for example, to evaluate anti-inflammatory therapeutics involving macrophages.
